# Elimination of a ligand gating site generates a supersensitive olfactory receptor

**DOI:** 10.1038/srep28359

**Published:** 2016-06-21

**Authors:** Kanika Sharma, Gaurav Ahuja, Ashiq Hussain, Sabine Balfanz, Arnd Baumann, Sigrun I. Korsching

**Affiliations:** 1 Institute of Genetics, Biocenter, University at Cologne, Zülpicherstrasse 47a, 50674 Cologne, Germany; 2 Institute of Complex Systems (ICS-4), Research Center Jülich, 52428 Jülich, Germany

## Abstract

Olfaction poses one of the most complex ligand-receptor matching problems in biology due to the unparalleled multitude of odor molecules facing a large number of cognate olfactory receptors. We have recently deorphanized an olfactory receptor, TAAR13c, as a specific receptor for the death-associated odor cadaverine. Here we have modeled the cadaverine/TAAR13c interaction, exchanged predicted binding residues by site-directed mutagenesis, and measured the activity of the mutant receptors. Unexpectedly we observed a binding site for cadaverine at the external surface of the receptor, in addition to an internal binding site, whose mutation resulted in complete loss of activity. In stark contrast, elimination of the external binding site generated supersensitive receptors. Modeling suggests this site to act as a gate, limiting access of the ligand to the internal binding site and thereby downregulating the affinity of the native receptor. This constitutes a novel mechanism to fine-tune physiological sensitivity to socially relevant odors.

The interaction of odors with their cognate receptors constitutes one of the most complex ligand/receptor binding problems in biology due to the sheer quantity of potential odor molecules facing a limited albeit huge number of different olfactory receptors which in some species comprise close to 10% of all proteins^1^,^2^. The tuning width of these receptors is extremely variable, with odor spectra ranging from exceedingly broad^3^,^4^ to monospecific^5^. In some cases a single functional group of the ligand dominates the specificity of the ligand/receptor interaction, in other cases an ensemble of chemical features is recognized^6^,^7^,^8^. However, only in very few cases do we possess a molecular understanding of the binding interaction between an odorant and its receptor^9^. So far crystal structures are not available for any olfactory receptor, and thus prediction of olfactory receptor structures has relied on modeling studies using established templates such as the beta-adrenergic receptor (β-AR)^10^ and rhodopsin^11^, further supported by site-directed mutagenesis and subsequent functional analysis of mutant receptors^12^.

We have used a similar approach to unravel the ligand interaction of a zebrafish olfactory receptor specific for aliphatic diamines, TAAR13c^6^. The trace amine associated receptor (TAAR) family is the only olfactory receptor family that is much larger in teleost fish compared to tetrapods, suggesting an essential role for TAARs in fish^13^. Zebrafish possess 112 *taar* genes, compared to only 15 in mouse and even less in the amphibian and avian lineages^6^. Since zebrafish serve as a model system for vertebrates, and their olfactory system is qualitatively similar to that of vertebrates including mammals^14^, zebrafish are well suited to gain deeper insight into vertebrate olfactory receptor properties.

We have recently shown TAAR13c to be a highly sensitive and specific receptor for the death-associated odor cadaverine^6^, which emanates from carrion *via* bacterial decarboxylation of lysine. Cadaverine is strongly repulsive for humans and, interestingly, it also elicits strong innate aversive behavior in zebrafish^6^. At low concentrations of cadaverine mostly TAAR13c-expressing neurons get activated suggesting that a single olfactory receptor might suffice to generate a powerful odor-driven behavior^6^. Here we aimed to understand the molecular basis at the very beginning of this neural circuit, i.e. the interaction of TAAR13c with its native ligand cadaverine.

We have performed thorough modeling of the TAAR13c receptor to identify potential binding site residues, and found all of them clustering in the upper third of the transmembrane domains of TAAR13c. We mutated several of these candidate residues and compared the activation of mutant to wildtype receptors in a heterologous cell expression system. Two aspartates, Asp112^3.32^ and Asp202^5.42^, buried in the plane of the membrane, were identified as essential components of an internal binding site for cadaverine. Another aspartate, Asp279^6.58^, was found to constitute an essential residue of a second binding site located at the extracellular surface of the receptor. Both conservative and non-conservative substitutions of Asp279^6.58^ generated supersensitive receptors. Based on our modeling data we suggest the external binding site to act as a gate, which cadaverine has to pass on its way to the internal binding site. As long as the external binding site is occupied, the gate is closed, and thus limits the free access of cadaverine to the internal binding site. This constitutes a novel molecular mechanism for regulating ligand access to the activating binding site. To the best of our knowledge, such a gating mechanism has not been suggested for any olfactory receptor of any species so far.

## Results

### Modeling of TAAR13c predicts the expected 7TM structure and two additional small helices in ECL2 and C-terminus

We have modeled the TAAR13c structure as a prerequisite to gain structural insights into the molecular architecture and functional constraints of its binding pocket. The homology model of TAAR13c was based on X-ray crystal structures of six templates (see Mat. & Meth and Supplementary Data 2). The TAAR13c primary structure shares a maximal identity of 33% to its closest homolog, the β_1_ adrenergic receptor (β_1_AR; Protein Data Bank Entry 4AMJ)^15^.

**Figure 1:**
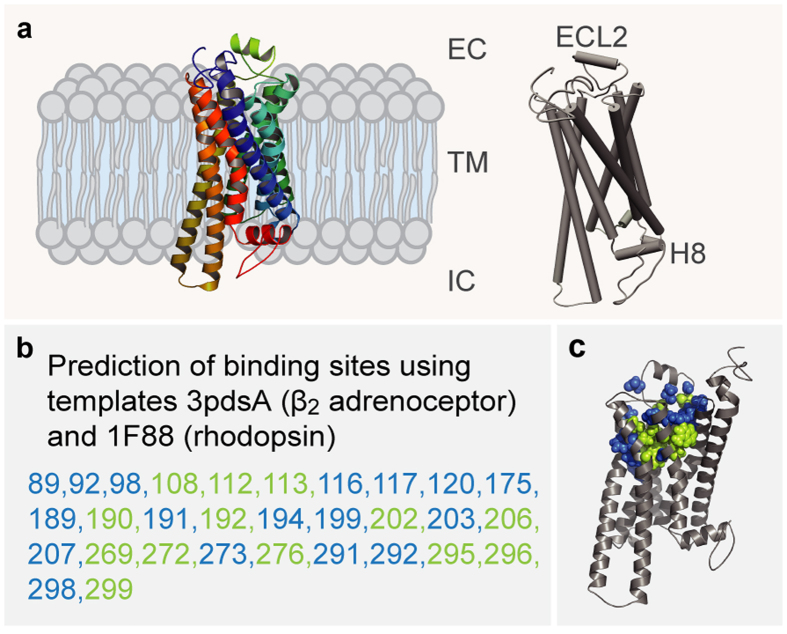
Homology modeling of TAAR13c predicts 7 TM, two additional short helices, and potential binding residues clustered in the upper third of the TM region. (**a**) Cartoon representation of the TAAR13c model based on comparison with six crystal structures shows the expected seven transmembrane domains and two short extra helices. The planks representation to the right shows a short α-helix to be located in ECL2 and an intracellular eighth helix, H8 located parallel to the membrane plane. (**b**) Ligand binding residues (given as residue number in TAAR13c) as predicted by sequence profile comparison with binding sites of PDB templates 3pdsA (FAUC-50-β_2_ adrenoceptor complex) and 1F88 (bovine rhodopsin); blue, residues only reported in one of the models; green, residues predicted in both models. (**c**) Predicted binding residues listed in panel (**b**) shown as spheres in the TAAR13c structure, color code as before. Note the presence of two ‘green’ columns.

The homology model of TAAR13c (Fig. 1a) revealed the canonical bundle of seven transmembrane (TM) α-helices followed by an eighth intracellular helix (H8) running parallel to the membrane axis. In rhodopsin, an interaction between H8 and TM7 keeps the receptor in a prereceptive state^16^, but no such interaction was seen in the TAAR13c model. Interestingly, in the model based on β_1_AR we also observed a short α-helix in extracellular loop 2 (ECL2) of TAAR13c, which is absent in most class A GPCRs, but present in the β-adrenergic receptors^17^. Furthermore, a disulfide bridge is predicted between Cys105^3.25^ at the extracellular end of TM3 and Cys190 in ECL2. This disulphide bond provides conformational restraint and is important for effectively tethering ECL2 to the helical bundle^18^. The highly conserved landmark motif DRH/Y, here DRH, is located at the cytoplasmic end of TM3 as expected^17^. This motif stabilizes the inactive state in some receptors, and governs G-protein coupling in other receptors^19^. We also observed the ‘ionic lock’ between the DRH motif and a glutamate residue at the cytosolic surface of TM6. Due to a salt bridge that is formed between Arg130^3.50^ of the DRH motif and Glu251^6.34^ in TM6, the third and sixth TM helices are connected, a feature which is conserved among all family A GPCRs^17^.

### Docking of cadaverine validates a binding pocket in the outer-third of the TM domain

We then used COACH^20^ to predict putative cadaverine binding residues by sequence profile comparison of TAAR13c with binding sites of several PDB structures, with best fits found for 3pdsA (FAUC50/β_2_ adrenoceptor complex) and 1F88 (bovine rhodopsin) in the TAAR13c homology model (Fig. 1b). COACH is a meta-server approach to generate complementary ligand binding site predictions using comparative methods, which recognize ligand-binding templates from BioLiP protein function database by binding-specific substructure and sequence profile comparisons^21^. Initially 30 such residues were found. They all clustered in the upper third of the TM domain suggesting that the putative binding pocket is located within this region (Fig. 1c). The classical amine-binding motif of aminergic receptors consists of Asp112^3.32^ and Trp296^7.40^, both of which were also predicted as binding partners of cadaverine^22^. In close proximity to these residues and at the same plane of the membrane another aspartate residue, Asp202^5.42^, was predicted as a binding partner. We hypothesized that this residue might be involved in binding to the second amino group of cadaverine and examined the region surrounding Asp112^3.32^, Asp202^5.42^ and Trp296^7.40^ (i.e. the upper one-third of TM3, 5, and 6) by computational docking of cadaverine.

**Figure 2:**
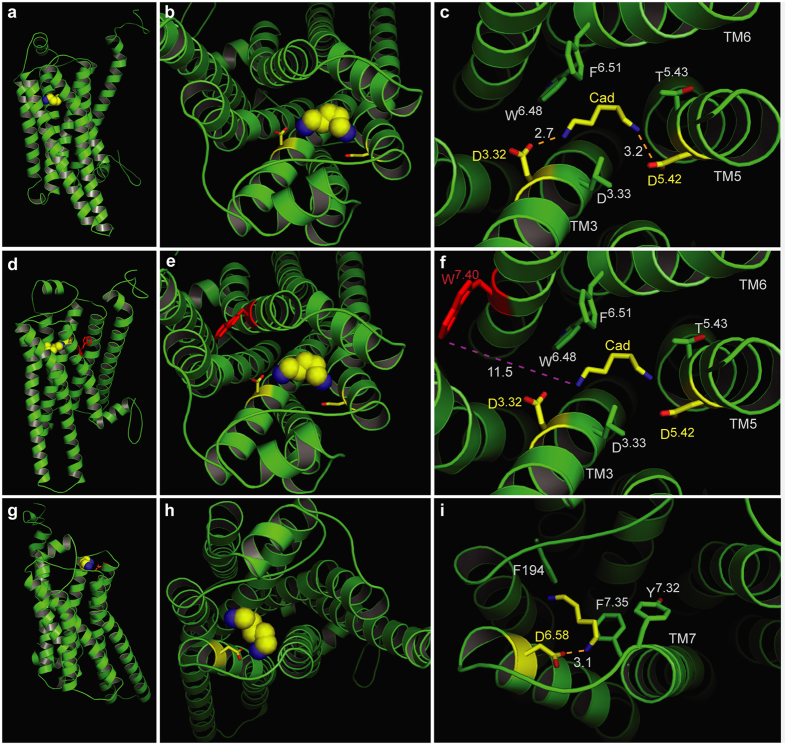
Two binding sites for cadaverine predicted by docking to wildtype TAAR13c. TAAR13c structure (green) is shown with cadaverine (cad; yellow, backbone; blue, amino groups) and coordinating aspartate residues (yellow, backbone; red, carboxyl group). (**a**) Sideview of TAAR13c shows spatial position of cadaverine docked to the internal binding site, located in the external third of the TM region. (**b**) Enlarged view from the extracellular surface onto the same binding site shown in panel (**a**). (**c**) Enlargement from panel (**b**) (same view) showing cadaverine, the major interacting residues and the distances in Å from the carboxyl groups of aspartates D^3.32^ and D^5.42^ to the amino groups of cadaverine. Salt bridges are visualized as orange dashed lines. (**d**) Side view (turned about 90° compared to panel (**a**) shows the side chain of W^7.40^ located away from the predicted binding site. (**e**) View from the extracellular surface, enlarged, same orientation as panel (**b**). W^7.40^ is positioned on the distal side of its TM relative to the binding site. (**f**) Enlargement from panel (**e**) (same view) showing cadaverine, the two interacting aspartate residues and the large distance to the side chain of W^7.40^ (purple dashed line), which is thus unlikely to participate in binding interactions. (**g**) Side view (turned about 90° compared to panel (**a**) showing cadaverine bound at an additional binding site on the external surface. (**h**) View onto the extracellular surface, enlarged, orientation turned 180° relative to panel (**b**). A single aspartate (D^6.58^) coordinates cadaverine. (**i**) Enlargement from panel (**h**) (similar view) showing cadaverine, the major interacting residues and the distance from the carboxyl group of D^6.58^ to the amino group of cadaverine. The salt bridge is visualized as orange dashed line.

The docking results confirmed the involvement of Asp112^3.32^ and Asp202^5.42^ in ligand binding. Our results suggest that one amino group of cadaverine (protonated at physiological pH) forms a salt bridge with Asp112^3.32^ at a distance of 2.7 Å (Fig. 2c, Table 1), well within the range given for salt bridges 1.75–4.0 Å^23^. This Asp112^3.32^ is stabilized by a hydrogen bond to the hydroxyl group of Tyr299. A similar salt bridge has been described for β_1_- and β_2_ARs^24^. The second protonated amino group of cadaverine was docked 3.2 Å away from Asp202^5.42^ allowing formation of another salt bridge (Fig. 2c). This particular residue is known to undergo binding interactions with ligands in many other GPCRs, forming hydrogen bonds, van der Waals interactions and salt bridges^25^. Further binding residues validated by docking were Leu113^3.33^, Thr203^5.43^, Trp269^6.48^, and Phe272^6.51^, all situated within 5 Å distance from cadaverine and thus well within the range of van der Waals interactions (3–6 Å^26^) (Supplementary Table 1). Thus, these residues are likely candidates for stabilizing the hydrophobic backbone of cadaverine. In addition to cadaverine, diaminoheptane, which has a very similar affinity to TAAR13c^6^, forms the same two salt bridges as cadaverine (2.9 Å distance to Asp112^3.32^ and 2.4 Å to Asp202^5.42^).

Docking did not confirm Trp296^7.40^ as a binding residue in TAAR13c. Notably, Trp296^7.40^ is 11.5 Å away from the docked cadaverine, and furthermore the residue is located on the distal side of TM7 relative to cadaverine, excluding a van der Waals interaction (Fig. 2f). This was unexpected, because in class A GPCRs this tryptophan is highly conserved, and serves to stabilize the hydrophobic backbone of amines as part of the amine-binding motif^22^.

In addition to cadaverine, TAAR13c is activated by putrescine, a smaller diamine, albeit with much lower affinity^6^. Docking of putrescine into the TAAR13c model revealed the same salt bridge with Asp112^3.32^ as for cadaverine, albeit at a slightly larger distance of 3.0 Å (Table 1). However, Asp202^5.42^ is not able to form the second salt bridge, because the distance of 5.5 Å between the amino group of putrescine and the carboxylic group of Asp202^5.42^ (Table 1) is too large for a typical salt bridge and only allows a rather weak binding interaction^27^. This finding would explain the decrease in affinity of TAAR13c for putrescine compared to cadaverine. Nevertheless the weak interaction between Asp202^5.42^ and the second amino group of putrescine seems to be relevant because the corresponding monoamine (butylamine) is not able to activate TAAR13c at all^6^.

Taken together, these data strongly suggest that TAAR13c activation relies on the interaction of two amino groups provided by the ligand with two negatively charged residues in the binding cavity of the receptor, and that stabilization of the ligand backbone is not achieved by the canonical tryptophan, here Trp296^7.40^, in TM7 of the DW motif.

### Docking of cadaverine suggests a second binding site for diamines

In addition to the above-mentioned residues, we identified another docking site on the extracellular surface of the receptor (Fig. 2g,h). The main binding residue of this second site is Asp279^6.58^, which forms a salt bridge with one amino group of cadaverine (Fig. 2i) and putrescine, each at a distance of 3.1 Å (Table 1). Surrounding apolar residues Phe194 in ECL2 and Phe291^7.35^ in TM7 are also predicted as interaction partners, and presumably serve to stabilize the ligand’s apolar backbone (Supplementary Table 1). No residue coordinating the second amino group was detected in this docking site, suggesting that it might not discriminate for chain length. Thus, this site differs in two properties from the internal docking site: (i) it does not noticeably distinguish between cadaverine and putrescine, and (ii) it does not require a second amino group to bind the ligand. Since TAAR13c activation strongly depends on the ligand’s chain length and absolutely requires the second positive charge of the ligand^6^, this second docking site containing Asp279^6.58^ is rather unlikely to serve as the ligand binding site that activates the receptor. In order to provide independent experimental proof for the predicted docking residues, we generated a series of receptor mutants by site-directed mutagenesis and studied receptor activity after heterologous expression in mammalian cells.

### Mutation of the aminergic DW motif shows only the aspartate as required for ligand binding

**Figure 3:**
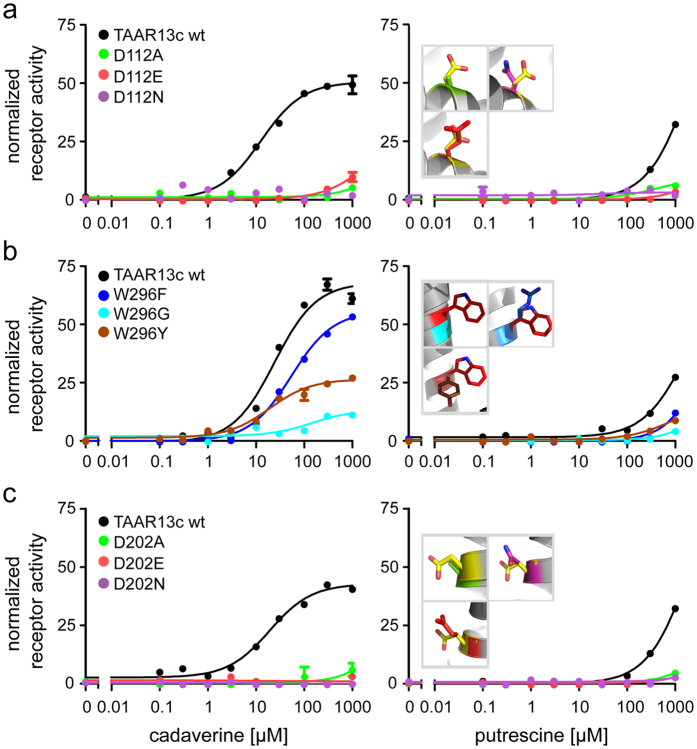
Concentration-response curves of TAAR13c mutated in the internal binding site and Trp296^7.40^. HEK293 cell lines constitutively expressing CNG channels and either wildtype or TAAR13c mutants were incubated with concentration series of cadaverine or putrescine (10 nM to 1 mM). Changes in intracellular Ca^2+^ were detected by Fluo-4 and calculated as ∆F/F. Values were normalized to the fluorescence ratio obtained with NKH477, an agonist of membrane-bound adenylyl cyclases. Representative binding curves are shown color-coded for mutation (green, A; red, E; magenta, N; blue, F; cyan, G; brown, Y), error bars (SD, n = 4) are shown if exceeding symbol size. Left column, responses to cadaverine; right column, responses to putrescine. Insets in right panels show the positions of the mutant side chains (same color as for the respective binding curves) overlayed over the wildtype residue (D, yellow and W, red, respectively). (**a**) Mutation of the D112 residue to D112E, D112N and D112A results in almost complete loss of activation by cadaverine, with small residual activity only at the highest concentration tested, 1mM. Activation by putrescine is abolished in all D112 mutants. (**b**) Mutants W296F, W296Y and even W296G were activated by both cadaverine and putrescine, at similar EC50’s as wildtype TAAR13c. Efficacy for W296F is very similar to wildtype TAAR13c but reduced for W296Y and W296G. (**c**) Mutation of the D202 residue to D202E, D202N, and D202A results in complete loss of activation by cadaverine and putrescine.

As described above, docking predicted only the Asp112^3.32^ but not the Trp296^7.40^ residue of the conserved DW motif to interact with cadaverine (Fig. 2) and putrescine. A series of substitutions were generated for both residues to examine their effects on receptor activity. For the aspartate we chose D112E as a conservative exchange, since the charge is kept and only its position is slightly changed due to the longer side chain in glutamate. Other mutations employed were D112N, which eliminates the charge, but keeps the polarity, and finally D112A, which removes both the charge and polarity. Mutant receptors were stably transfected into HEK293 cells that constitutively express the A2 subunit of an olfactory cyclic nucleotide-gated (CNG) ion channel (see Mat. & Meth.). Activation of TAAR receptors and subsequent cAMP production could be monitored in these cell lines as elevated Ca^2+^ levels due to Ca^2+^ influx through cAMP-dependent opening of the CNG channels. We observed that even the most conservative exchange, D112E, reduced TAAR13c activation drastically, and shifted the dose response curve for cadaverine more than two orders of magnitude to higher concentrations (Fig. 3a, Table 1). Mutation to either asparagine or alanine completely abolished cadaverine-evoked activity. Consistent with these experimental results, docking simulations with cadaverine showed the absence of the wildtype salt bridge in all three mutants (Table 1). Taken together we conclude that Asp112^3.32^ is a pivotal part of the binding site leading to activation of the TAAR13c receptor by cadaverine.

Similar effects were observed for the mutant receptors when putrescine was applied as a ligand (Fig. 3a, Table 1), and again, even the D112E variant displayed a drastic reduction of the putrescine response. These results confirm the involvement of Asp112^3.32^ also for the predicted interaction with putrescine. We were intrigued that binding to putrescine, which is one methylene group shorter than cadaverine, could not be improved by the longer side chain of glutamate^28^, and therefore performed docking simulations with the D112E mutant and putrescine. Indeed, the distance between the charged glutamate side chain and the amino group of putrescine was predicted as 9.8 Å (Table 1), well beyond the range of a salt bridge^27^, and thus consistent with the drastic reduction in affinity observed for the D112E receptor mutant. In fact, docking of putrescine showed for all three mutants that at most one amino group is able to form a typical salt bridge, whereas the other amino group is too far away for a binding interaction (Table 1). Taken together, experimental and modeling results obtained with Asp112^3.32^ mutants and putrescine again confirmed the involvement of Asp112^3.32^ in ligand binding as predicted by modeling and docking the wildtype receptor (see above).

The tryptophan residue Trp296^7.40^ of the DW motif^13^ was not predicted to be part of the binding site (Fig. 2f). We therefore examined whether replacement of this residue might affect receptor activity. Three mutants were generated, either exchanging the tryptophan for phenylalanine (less bulky), tyrosine (switch to polar residue) or glycine (no side chain interaction possible). Stably transfected cell lines were established with all mutants, and receptor activity was examined with a series of cadaverine and putrescine concentrations (Fig. 3b).

Cadaverine and putrescine were able to activate all three mutants, with similar affinity as wildtype, even for the drastic W296G exchange (Fig. 3b, Table 1), in sharp contrast to the almost complete loss of activity in all Asp112^3.32^ mutants. Furthermore, the efficacy of W296F was similar to wildtype TAAR13c. A slight reduction of efficacy was observed for the W296Y mutant, which might be caused by a different interaction of the more hydrophilic tyrosine with neighboring side chains compared to the tryptophan in wildtype TAAR13c. Efficacy was strongly reduced in the W296G mutant, conceivably due to a loss of structural stability by insertion of the highly flexible glycine into a transmembrane domain. Taken together, binding experiments for all three mutants, W296F, W296Y, and W296G, show little loss in affinity and (with one exception) efficacy, confirming that Trp296^7.40^ is irrelevant for the binding interaction to cadaverine and putrescine, as predicted by the theoretical model.

### A second aspartate, Asp202^5.42^ in TM5 is required for activation of TAAR13c by cadaverine and putrescine

Docking studies suggested an interaction of Asp202^5.42^ with the second amino group of cadaverine (Fig. 2c). We followed a similar strategy as before and generated cell lines expressing the following receptor mutants: D202E, D202N, and D202A. Functional testing with cadaverine revealed that even the most subtle replacement, the D202E substitution, eliminated the activity of the receptor completely (Fig. 3c). The same results were obtained for the other substitutions of D202 to either asparagine or alanine (Fig. 3c). Docking simulations for cadaverine with the mutants showed a large distance of about 10 Å between the mutated residues and the second amino group of cadaverine, consistent with a loss of the second salt bridge present in the wildtype receptor (Table 1). Hence we conclude that Asp202^5.42^ is another pivotal residue in the cadaverine binding site and participates in the activation of the TAAR13c receptor.

As observed for cadaverine, all three receptor variants remained quiescent when cell lines were treated with putrescine (Fig. 3, Table 1). Docking data showed that the distance between the glutamate and the second amino group of putrescine was about twice as large as in the wildtype receptor (Table 1), consistent with the inability of putrescine to activate the D202E mutant receptor. A similar result was obtained for the other two mutations (Table 1). Thus, all experiments performed with Asp202^5.42^ mutants are consistent with docking results for these mutants, and confirm the prediction of Asp202^5.42^ as an essential component of the diamine binding site that can activate TAAR13c.

### Elimination of the external binding site for diamines generates a supersensitive receptor

**Figure 4:**
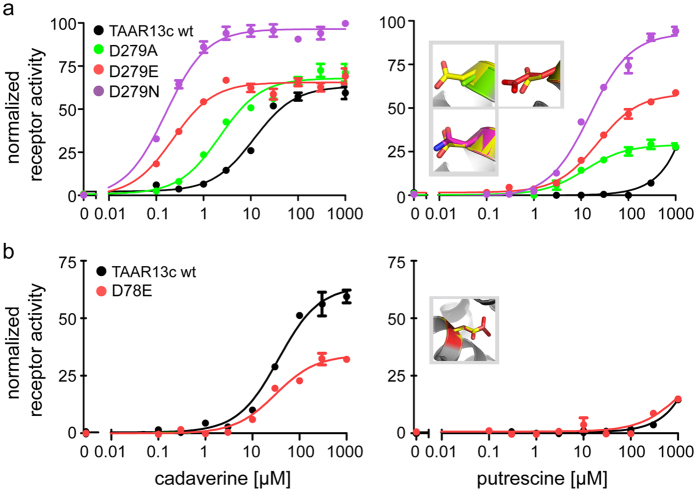
Concentration-response curves of TAAR13c mutated in the external binding site and a control site. HEK293 cell lines constitutively expressing CNG channels and either wildtype or TAAR13c mutants were incubated with concentration series of cadaverine or putrescine (10 nM to 1 mM). Changes in intracellular Ca^2+^ were detected by Fluo-4 and calculated as ∆F/F. Values were normalized to the fluorescence ratio obtained with NKH477, an agonist of membrane-bound adenylyl cyclases. Representative binding curves are shown color-coded for mutation (green, A; red, E; magenta, N; blue, F; cyan, G; brown, Y), error bars (SD, n = 4) are shown if exceeding symbol size. Left column, responses to cadaverine; right column, responses to putrescine. Insets in right panels show the positions of the mutant side chains (same color as for the respective binding curves) overlayed over the wildtype residue (D, yellow and W, red, respectively). (**a**) For mutant D279E the apparent affinity to cadaverine and putrescine as estimated by EC_50_ increased about twenty and hundredfold, respectively, compared to wildtype. EC_50_ values for binding of cadaverine to D279N and D279A mutants increased 14fold and 7fold, respectively. Notably, the EC_50_ values for binding of putrescine to D279N and D279A mutants were as low as those observed for wildtype TAAR13c binding to cadaverine. (**b**) Mutant D78E bound cadaverine and putrescine similarly well compared to wildtype. Since this aspartate residue is located in the TM region, but outside of the ligand binding site, this result shows that an exchange of aspartate to glutamate in the TM region *per se* does not impair receptor activity.

To examine the relevance of the potential binding site on the extracellular face of TAAR13c predicted by docking, we generated three variants of Asp279^6.58^, i.e. D279E, D279N, and D279A, and expressed them stably in cell lines. All three receptor variants displayed a massive increase in affinity and efficacy (Fig. 4a). The most pronounced effect was observed for D279E, whose apparent affinity (EC_50_) to cadaverine increased over twentyfold (Table 1). For the D279N and the D279A mutant receptors we observed a 14fold and a 7fold increase of EC_50_, respectively (Table 1). The efficacy for all three mutants increased as well, with the most pronounced effect seen for the D279N mutant - about twice the value of wildtype TAAR13c (Fig. 4a). Hence modifications of Asp279^6.58^ result in supersensitive receptors. A similar increase in affinity and efficacy of these three receptor variants was measured when cell lines were treated with putrescine (Fig. 4a, Table 1). Notably, EC_50_’s of the mutants for putrescine reached values of 7–30 μM, very similar to those determined for cadaverine on wildtype TAAR13c.

When we performed docking simulations, no external binding site was predicted for all three mutants, i.e. the modification of Asp279^6.58^ eliminated this binding site. The internal binding site, however, remained intact, with distances to the ligand amino groups in the range of those observed for wildtype TAAR13c (Table 1), and similar subsets of additional contact sites (Supplementary Table 1).

These observations are consistent with the hypothesis that a ligand-occupied external binding site blocks access to the internal binding site. Elimination of the external binding site relieves this block, leading to an increase in effective ligand concentration in the vicinity of the internal binding site, thus generating a supersensitive receptor.

## Discussion

It is a complex but fascinating challenge to gain deeper understanding how the binding of a multitude of different odor molecules to a large cohort of olfactory receptors evokes specific cellular responses. In recent years ligands have been reported for many olfactory receptors^4^,^6^,^29^,^30^. However, only in very few cases do we possess a molecular understanding of the binding interaction between an odorant and its receptor^9^. So far crystal structures are not available for any olfactory receptor, and thus prediction of olfactory receptor structures has relied on modeling studies. Here we used modeling and docking to predict binding interactions of a receptor linked to a robust innate avoidance behavior. This receptor is a member of the TAAR family of olfactory receptors, TAAR13c, which is activated by the death-associated odor cadaverine in the micromolar range, and, much less efficiently, by the closely related diamine putrescine^6^.

We applied a two-pronged approach to elucidate the mechanism of TAAR13c activation: (i) receptor modeling and docking to uncover residues participating in ligand binding and receptor activation; (ii) site-directed mutagenesis of predicted binding residues followed by functional analysis in an heterologous expression system. For wildtype TAAR13c and all mutants we performed docking simulations with cadaverine and putrescine, and determined bond length between pivotal binding residues of the receptor and ligand amino groups as an estimate of binding strength. In all cases predicted alterations in bond lengths of the mutant receptors were consistent with the experimentally observed changes in activity profiles.

Our results identified Asp112^3.32^ and Asp202^5.42^, but not Trp296^7.40^ as essential elements of a binding site for cadaverine and putrescine in the upper third of the TM region (internal binding site). The results for the mutation of the two aspartates to alanine reaffirm a recent independent study^31^. Our broader approach to the mutational analyses including the conservative exchanges from aspartate to glutamate and asparagine allows us to confidently state that the observed loss of receptor activation is not due to general conformational changes caused by the rather drastic aspartate to alanine exchange. Furthermore, we also examined whether an aspartate to glutamate transition as such could impair receptor function. We mutated an aspartate residue within the TM region of the receptor, but outside of the predicted binding site (D78^2.50^) to glutamate. This mutation did not impair the receptor’s properties, strongly suggesting that the exchange of an aspartate to glutamate in the TM region as such is not sufficient to generate coarse conformational changes resulting in a loss of receptor function (Fig. 4b). Similarly, mutations of Trp296^7.40^ did not alter the receptor’s activation profile by cadaverine and putrescine. Thus, the drastic changes in TAAR13c affinity seen even for the conservative D112E and D202E exchanges most likely are due to the structural change within the binding pocket. The requirement for binding residues for two functional groups we reported here is fully consistent with the ligand profile of TAAR13c^6^.

Asp112^3.32^ and Trp296^7.40^ together constitute the so-called DW motif, which is highly conserved in aminergic receptors^25^,^32^. Asp112^3.32^ is assumed to coordinate the amino group of amine-containing ligands, and Trp296^7.40^ is supposed to interact with the apolar or aromatic backbone. However, the orientation of the diamines cadaverine and putrescine in the binding pocket necessitated by coordination of the second amino group to Asp202^5.42^ precludes coordination of the backbone by Trp296^7.40^, in accordance with the experimental results.

Interestingly, a second binding site for cadaverine and putrescine was predicted at the external surface of TAAR13c, right above the internal binding site discussed in the preceding paragraph. Mutation of the pivotal element of this binding site, Asp279^6.58^, resulted in a supersensitive receptor, which displayed an increase in apparent affinity (EC_50_) for cadaverine and putrescine of up to two orders of magnitude. This rather unexpected result prompted us to examine the predicted structure of TAAR13c for possible mechanistic explanations. Both the internal and the external binding site were predicted by modeling and confirmed by mutational analysis as discussed above. However, whereas the internal binding site (Asp112^3.32^, Asp202^5.42^) is necessary for receptor activation, the external binding site (Asp279^6.58^) is not, and in fact impairs receptor activation.

**Figure 5:**
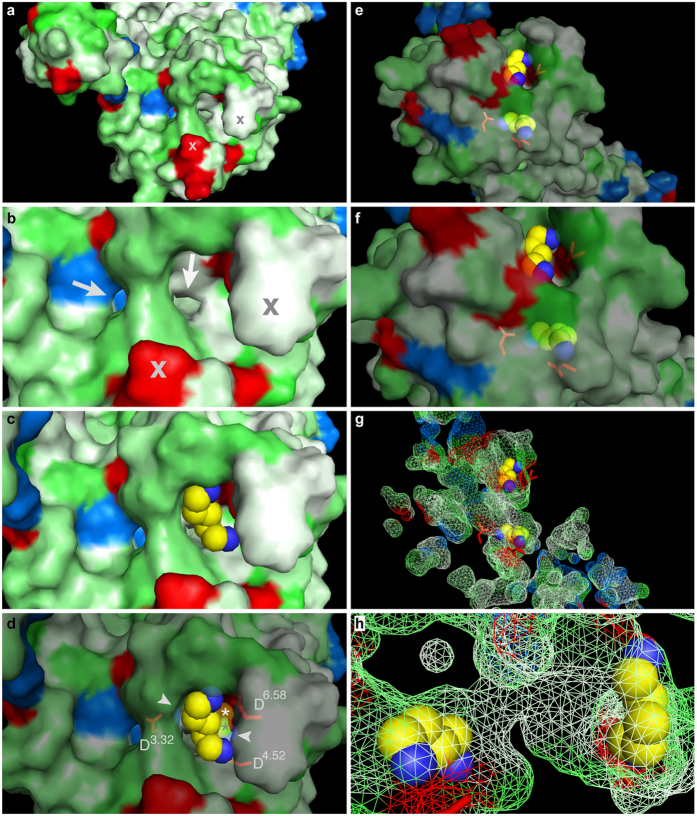
Occupied external binding site blocks access of cadaverine to the interior binding and activation site. Surface view (panels **a**–**f**) and mesh view (panels **g**,**h**) of the TAAR13c model docked with cadaverine visualizes a potential access path for cadaverine. (**a**) View from the extracellular surface; red, acidic residues; blue, basic residues; green, polar residues; white, hydrophobic residues; cross, same protrusions as marked in panel (**b**). (**b**) Enlarged detail of panel a; arrows point to two potential access paths for passage of cadaverine to the internal binding site. Note that the positively charged Arg92^2.64^ precludes access via left route. (**c**) Cadaverine docked into the external binding site, same view as in panel b. Note that the access to the internal binding site is blocked. (**d**) Same view as panel (**b**,**c**), but partly transparent; aspartates of external (D^6.58^) and internal (D^3.32^ and D^4.52^) binding sites are shown in stick mode; asterisks mark cadaverine bound to the internal binding site; arrowheads point to the amino groups of cadaverine docked into the internal binding site, which lies right below the external binding site shown in top view. (**e**) Slightly tilted view onto the external surface to display both binding sites at once, external binding site is up. Partial transparency visualizes cadaverine docked into the internal binding site, down; aspartates of external and internal binding sites are shown in stick mode, red. (**f**) Enlargement of panel (**e**). (**g**) Same orientation and magnification as in panel e; mesh view of all predicted cavities, with cadaverine docked into the external and internal binding site. (**h**) Enlarged view turned 90° counter clockwise and 90° front-to-back compared to panel (**g**). Note the tunnel connecting the external (left) and internal (right) binding site.

We hypothesized that the external binding site, when occupied, might block access of the ligand to the internal binding site. There are only two conceivable access points to the internal binding site, highlighted in the surface view of the docking model (Fig. 5a,b). One of these sites is flanked by a positively charged residue, Arg92^2.64^. This constraint makes it unlikely to allow passage of positively charged diamine compounds into the receptor’s internal binding pocket.

The second access point has no such restrictions and thus appears suitable to allow access of the ligand to the internal binding site (Fig. 5b,h). This access point is located directly below the external binding site and thus can be expected to be blocked as long as the external binding site is occupied by ligand (Fig. 5b,c). Only after dissociation of the ligand from the external binding site there is a chance for it to travel towards the internal binding site (Fig. 5h). Thus the external binding site acts as a gate that may be closed (binding site occupied by ligand) or open (binding site not occupied). Elimination of the external binding site by exchanging Asp279^6.58^ destroys the gate and ligand access to the internal binding site is no longer impeded by the intermediary step of binding to the external site. Thus, a supersensitive receptor is generated. In the wildtype TAAR13c, the external binding site serves to downregulate the affinity to diamines, conceivably to adjust it to a physiologically meaningful range. This phenomenon constitutes a novel mechanism in olfactory receptor function.

Some recent reports in the literature describe increases in affinity for mutations of the ligand binding site that leads to receptor activation^33^,^34^. These effects are generally small (often two fold, in rare cases up to tenfold). In contrast, here we report a considerably stronger effect on receptor affinity - up to two orders of magnitude - by introducing mutations far away from the activating binding site, and also far away from the intracellular signal transducing regions. Downregulation of a receptor’s activity by occupation of an external ligand binding site constitutes a distinct, novel mechanism, by which odorant access to the internal ligand binding site that leads to receptor activation is impaired. Future studies will be required to elucidate, whether such a mechanism might occur in perception of other socially relevant odors, and how widespread it might be in olfactory receptor activation.

During evolution, the affinity of olfactory receptors had to be constantly tuned to physiologically relevant odor concentrations. Sometimes this may have amounted to downtuning the affinity, either directly at the activating binding site, or indirectly *via* a ligand-regulated gating mechanism such as described here for TAAR13c. Gating mechanisms to regulate open/closed states of ion channels and transmitter receptors are well known. Here we show for the first time that gating also plays a role in olfactory perception.

## Materials and Methods

### Heterologous expression of TAAR13c receptor mutants

Cell lines that constitutively expressed either the wildtype TAAR13c receptor or a receptor mutant were generated using a previously established protocol^35^. We used a cell line that had been stably transfected with a gene encoding a variant of the A2-subunit of the olfactory cyclic nucleotide-gated (CNG) ion channel^36^,^37^. Approximately 8 μg of the respective TAAR13c expression vectors were introduced into ~4 × 10^5^ cells by a modified calcium-phosphate method^38^. Stably transfected cells were selected in the presence of the antibiotic G418 (0.8 mg/ml). Expression of TAAR13c was monitored by Western blotting (Supplementary Data 1) with specific anti-TAAR13c antibodies^6^ and anti-Rhodopsin antibodies (Sigma Aldrich, Taufkirchen, Germany).

### Monitoring functional TAAR13c receptor activity in cell lines

Activation of TAAR13c by cadaverine and putrescine evoked a rise in intracellular cAMP concentration that activates the CNG channels^35^ and thereby causes an influx of Ca^2+^ through the open channels. Changes in [Ca^2+^]_i_ were monitored with the Ca^2+^-sensitive fluorescent dye Fluo-4. Cells were grown in 96-well dishes to a density of approximately 2 × 10^4^ cells per well. Cells were loaded at room temperature with Fluo-4 AM as described previously^37^. After 90 min, the loading solution was substituted for dye-free ECS (extracellular solution; 120 mM NaCl, 5 mM KCl, 2 mM MgCl_2_, 2 mM CaCl_2_, 10 mM HEPES, and 10 mM glucose, pH 7.4 [NaOH]) containing 100 μM IBMX. The plate was transferred into a fluorescence reader (FLUOstar Omega, BMG Labtech, Offenburg, Germany) to monitor Fluo-4 fluorescence. The excitation wavelength was 485 nm. Fluorescence emission was detected at 520 nm. A concentration series of cadaverine or putrescine (10^−7^ to 10^−3 ^M) as well as 10^−5 ^M NKH477 (positive control) was added once Fluo-4 fluorescence had reached a stable value in each well. The changes in Fluo-4 fluorescence were recorded automatically. The fluorescence signal generated by the adenylyl cyclase activator NKH477 was set to 100% as an internal standard. Concentration–response curves were established from at least three independent experiments with quadruplicate measurements in each experiment.

Data were analyzed and displayed using Prism 5.04 software (GraphPad, San Diego, CA, USA).

### Introduction of mutations into TAAR13c

As starting point for mutagenesis we used a full length TAAR13c cDNA construct that harbored an N-terminal extension encoding the first 20 amino acids of bovine rhodopsin in pcDNA3.1 (−) expression vector^6^. Point mutations were introduced using the QuikChange Site-directed mutagenesis kit (Agilent Technologies, Santa Clara, USA). In brief, PCR reactions were performed using *PfuUltra* High-Fidelity DNA Polymerase with the above described plasmid as a template, and mutagenic primers listed in Table 2. Parental strands, which are methylated in contrast to the PCR products, were selectively digested with Dpn1 enzyme and the resulting product was transformed into XL-1 blue supercompetent *E.coli* cells by electroporation. Screening of recombinants was done by colony PCR using wild type TAAR13c primers. Colonies positive for the desired mutation were grown under standard conditions in LB broth and the plasmids were isolated using a plasmid DNA purification kit from Zymo research (California, USA). All mutations were verified by DNA sequencing.

### Homology modeling and ligand docking of TAAR13c

Homology models of TAAR13c were generated using GPCR-I-TASSER^39^ based on the crystal structure of six homologous templates and sequence alignments of these templates with TAAR13c (Supplementary Data 2). The sequence alignments were verified by inspection for proper aligning of conserved motifs and disulfide bridges. The model with the highest C-score (−0.36), which is well within the confidence range^40^, was chosen as the final structure. In initial experiments the mutant structure was generated with the above protocol and compared to a mutant model generated using side chain substitution in the wildtype structure using Chimera^41^. We observed no detectable RMSD difference (<0.05) between models generated by these two methods. Thus, subsequently homology models for mutants were generated by side chain substitution in the wildtype homology model. The receptor model was prepared for docking by adding protons and charges were assigned to ionizable side chains using the DockPrep function in Chimera. The model was then subjected to minimization using ModRefiner^42^. The final model backbone conformation was inspected by Ramachandran plot using Rampage^43^ (Supplementary Fig. 1). The ligand was used with flexible, rotatable bonds and was docked into the binding site using Autodock Vina^44^ with default parameter setting (iterated local search global optimizer)^44^. The resulting conformations were analyzed using AutoDock Tools^45^. PyMol^46^ was used for visualization of various ligand conformations and for preparing figures.

## Additional Information

**How to cite this article**: Sharma, K. *et al.* Elimination of a ligand gating site generates a supersensitive olfactory receptor. *Sci. Rep.* **6**, 28359; doi: 10.1038/srep28359 (2016).

## Supplementary Material

Supplementary Information

## Figures and Tables

**Table 1:** EC50 and contact distances for cadaverine and putrescine amino groups with Asp112^3.32^ and Asp202^5.42^ for mutant and wildtype TAAR13c.

Wildtype/Mutant	EC50 (μM, mean+/−SEM)		Distance to Cad-NH_2_		Distance to Put-NH_2_	
	cad	put	pos112	pos202	pos112	pos202
TAAR13c	15.2 ± 2.2	>1000	2.7	3.2	3.0	5.5
D112A	loss	loss	2.4	10.8	10.7	4.9
D112E	≫1000	loss	9.2	3.2	9.8	5.0
D112N	loss	loss	12.0	3.1	12.0	3.1
D202A	loss	loss	2.4	10.8	10.7	4.9
D202E	loss	loss	9.2	3.2	9.8	5.0
D202N	loss	loss	3.0	9.5	2.5	10.6
D279A	2.1 ± 1.0	31 ± 13	3.0	3.7	3.0	6.4
D279E	0.72 ± 0.24	10 ± 4	2.9	3.5	3.0	5.6
D279N	1.1 ± 0.8	7.3 ± 3.6	2.9	3.5	3.0	5.6
W296F	42 ± 6	>1000	–	–	–	–
W296G	55 ± 48	loss	–	–	–	–
W296Y	28 ± 8	>1000	–	–	–	–
D78E	64 ± 28	>1000	–	–	–	–

**Table 2:** Sequences of mutagenic primers used.

Wildtype/Mutant	Forward Primer, 5′-3′orientation	Reverse Primer, 5′-3′ orientation
TAAR13c	atggatttatcatcacaagaa	tcaaaccgtaaataaattgat
D112A	ccggttttgccctgtttctcac	gtgagaaacagggcaaaaccgg
D112N	acaccggttttaacctgtttctcac	gtgagaaacaggttaaaaccggtgt
D112E	ccggttttgaactgtttctcac	gtgagaaacagttcaaaaccgg
D202A	tggtcagttttagccacattacta	tagtaatgtggctaaaactgacca
D202N	tggtcagttttaaacacattacta	tagtaatgtgtttaaaactgacca
D202E	tggtcagttttagagacattacta	tagtaatgtctctaaaactgacca
D279A	actctctggtggctccctacattaac	gttaatgtagggagccaccagagagt
D279N	actctctggtgaatccctacattaac	gttaatgtagggattcaccagagagt
D279E	actctctggtggagccctacattaac	gttaatgtagggctccaccagagagt
W296G	tgatgcatttggtgggttaggctacac	gtgtagcctaacccaccaaatgcatca
W296Y	tgatgcatttggttacttaggctacac	gtgtagcctaagtaaccaaatgcatca
W296F	tgatgcatttggtttcttaggctacac	gtgtagcctaagaaaccaaatgcatca
D78E	ctggctctggcggaactgctgg	ccagcagttccgccagagccag
