# Supplementary material for: Elimination of a ligand gating site generates a supersensitive olfactory receptor
Source: Sci Rep. 2016 Jun 21;6:28359. doi: 10.1038/srep28359 (PMC4914996; doi:10.1038/srep28359)
Supplement: Supplementary Information [file srep28359-s1.doc]

# Elimination of a ligand gating site generates a supersensitive olfactory receptor

Kanika Sharma^1^, Gaurav Ahuja^1^*, Ashiq Hussain^1,2^*, Sabine Balfanz^3^*, Arnd Baumann^3^, Sigrun I. Korsching^1^

^1^ Institute of Genetics, Biocenter, University at Cologne, Zülpicherstrasse 47a, 50674 Cologne, Germany

^2^ Current address: Max Planck Institute for Neurobiology, Am Klopferspitz 18A, 82152 Martinsried, Germany

^3^ Institute of Complex Systems (ICS-4), Research Center Jülich, 52428 Jülich, Germany

* equal contribution

Corresponding author’s email sigrun.korsching@uni-koeln.de

# Supplementary Data 1 Western Blot analysis

Membrane proteins were prepared from parental (flpTM) and TAAR13c-expressing cells by standard methods. Briefly, cells were hypotonically lysed in a buffer consisting of 10 mM NaCl, 25 mM HEPES, pH 7.5, 2 mM EDTA, and protease inhibitor cocktail (Sigma Aldrich). After centrifugation, the pellet was solubilized in 100 mM NaCl, 25 mM HEPES, pH 7.5, 1% (w/v) CHAPS and protease inhibitor cocktail. Proteins were separated on 12.5% SDS-polyacrylamide gels and transferred by electro-blotting onto Immobilon-P PVDF membranes (Millipore, Schwalbach, Germany) using a semi-dry blotting apparatus following the manufacturers protocol (Sigma Aldrich, Taufkirchen, Germany). Membranes were blocked in 5% (w/v) low-fat dry milk in PBS buffer (PBS = 130 mM NaCl, 7 mM Na_2_HPO4 x 2H_2_O, 3 mM NaH_2_PO_4_ x H_2_O) and then incubated with affinity-purified anti-TAAR13c ^1^ dilution 1:1000 of 0.72 mg/ml stock solution or anti-Rhodopsin (mouse monoclonal antibody, Sigma Aldrich), dilution 1:1,000 in PBT (= PBS containing 0.02% (w/v) Tween 20). Membranes were washed with PBT and incubated with HRP-conjugated anti-rabbit or anti-mouse secondary antibodies (Sigma Aldrich; dilution 1:40,000 in PBT). After several successive rinses with PBT and PBS, immunoreactive bands were detected with an ECL-Kit (Applichem, Darmstadt, Germany).

# Supplementary Data 2 Templates for homology modeling and their alignment

a) The following templates were used for homology modeling of TAAR13c by GPCR-iTASSER ^2^ 3sn6R, 4amjA, 2rh1A, 4iarA, 4eiyQ, and 3zpqA.

b) Alignments of all six templates with TAAR13c generated in GPCR-I-TASSER; H is Helix; C is coil.

CCCCCCCCCCCCCCCCCCCCCCCCCCCCHHHHHHHHHHHHHHHHHHHHHHHHHCCCCCCC

Dr_Taar13c MDLSSQEYDPSQFCFPAVNNSCLKGTHHVSTQTVVYLILASAMTVTVLGNSVVIISIAHF 3sn6 ----------------------------VSTQTVVYLILASAMTVTVLGNSVVIISIAHF

2rh1 ---------------------------HVSTQTVVYLILASAMTVTVLGNSVVIISIAHF 4amj ---------------------------HVSTQTVVYLILASAMTVTVLGNSVVIISIAHF 4eiy ------------------------GTHHVSTQTVVYLILASAMTVTVLGNSVVIISIAHF

3zpqA ----------------------LKGTHHVSTQTVVYLILASAMTVTVLGNSVVIISIAHF 4iar --------------------NCLKGTHHVSTQTVVYLILASAMTVTVLGNSVVIISIAHF

CCCCCCHHHHHHHHHHHHHHHHHHHHHHHHHHHHHHHCCCCHHHHHHHHHHHHHHHHHHH

Dr_Taar13c KQLQTPTNILVMSLALADLLLGLVVMPFSMIRSVDGCWYYGETFCLLHTGFDLFLTSVSI 3sn6 KQLQTPTNILVMSLALADLLLGLVVMPFSMIRSVDGCWYYGETFCLLHTGFDLFLTSVSI 2rh1 KQLQTPTNILVMSLALADLLLGLVVMPFSMIRSVDGCWYYGETFCLLHTGFDLFLTSVSI 4amj KQLQTPTNILVMSLALADLLLGLVVMPFSMIRSVDGCWYYGETFCLLHTGFDLFLTSVSI 4eiy KQLQTPTNILVMSLALADLLLGLVVMPFSMI--VDGCWYYGETFCLLHTGFDLFLTSVSI 3zpqA KQLQTPTNILVMSLALADLLLGLVVMPFSMIRSVDGCWYYGETFCLLHTGFDLFLTSVSI 4iar KQLQTPTNILVMSLALADLLLGLVVMPFSMIRSVDGCWYYGETFCLLHTGFDLFLTSVSI

HHHHHHHHHHHHCCCCCCCCCCCCCHHHHHHHHHHHHHHHHHHHHHHHHHCCCCCCCCCC

Dr_Taar13c FHLIFIAVDRHQAVCFPLQYPTRITIPVAWVMVMISWSMAAFYSYGVVYSKANLEGLEEY 3sn6 FHLIFIAVDRHQAVCFPLQYPTRITIPVAWVMVMISWSMAAFYSYGVVYSKANLEGLEEY 2rh1 FHLIFIAVDRHQAVCFPLQYPTRITIPVAWVMVMISWSMAAFYSYGVVYSKANLEGLEE- 4amj FHLIFIAVDRHQAVCFPLQYPTRITIPVAWVMVMISWSMAAFYSYGVVYSKANLEGLEEY 4eiy FHLIFIAVDRHQAVCFPLQYPTRITIPVAWVMVMISWSMAAFYSYGVVYSKANLEGLEEY 3zpqA FHLIFIAVDRHQAVCFPLQYPTRITIPVAWVMVMISWSMAAFYSYGVVYSKANLEGLEEY 4iar FHLIFIAVDRHQAVCFPLQYPTRITIPVAWVMVMISWSMAAFYSYGVVYSKANV------

CCCCCCCCCCCCCCCCHHHHHHHHHHHHHHHHHHHHHHHHHHHHHHHHHHHCCCCCCCCC

Dr_Taar13c IASVYCMGGCTLYFNALWSVLDTLLTFFLPCSVMVGLYARIFVVAKKHIKSITEANQNEN 3sn6 ---IACMGGCTLYFNALWSVLDTLLTFFLPCSVMVGLYARIFVVAKKHIKSITEANQN-- 2rh1 --SVYCMGGCTLYFNALWSVLDTLLTFFLPCSVMVGLYARIFVVAKKHIKSITEANQNEN 4amj IASVYCMGGCTLYFNALWSVLDTLLTFFLPCSVMVGLYARIFVVAKKHIKSITE---NEN 4eiy IASVYCMGGCTLYFNALWSVLDTLLTFFLPCSVMVGLYARIFVVAKKHIKSITEANQNEN 3zpqA IASVYCMGGCTLYFNALWSVLDTLLTFFLPCSVMVGLYARIFVVAKKH------------ 4iar --------GCTLYFNALWSVLDTLLTFFLPCSVMVGLYARIFVVAKKHIKSITEANQNEN

CCHHHHHHHHHHHHHCCCCCCHHHHHHHHHHHHHHHHHHHHCCCCCCHHHHHHHHHHHHH

Dr_Taar13c ENVFKNPRRSERKAAKTLGIVVGAFILCWLPFFINSLVDPYINFSTPYALFDAFGWLGYT 3sn6 -------RRSERKAAKTLGIVVGAFILCWLPFFINSLVDPYINFSTPYALFDAFGWLGYT 2rh1 ENVFKNPRRSERKAAKTLGIVVGAFILCWLPFFINSLVDPYINFSTPYALFDAFGWLGYT 4amj ENVFKNPRRSERKAAKTLGIVVGAFILCWLPFFINSLVDPYINFSTPYALFDAFGWLGYT 4eiy ENVFKNPRRSERKAAKTLGIVVGAFILCWLPFFINSLVDPYINFSTPYALFDAFGWLGYT 3zpqA ---FKNPRRSERKAAKTLGIVVGAFILCWLPFFINSLVDPYINFSTPYALFDAFGWLGYT 4iar ENVFKNPRRSERKAAKTLGIVVGAFILCWLPFFINSLVDPY---STPYALFDAFGWLGYT

HHHHHHHHHHHCCHHHHHHHHHHHCCCCCCCCCCCCCCCCC

Dr_Taar13c NSTLNPIIYGLFYPWFRKTLSLIVTLRIFEPNSSDINLFTV

3sn6 NSTLNPIIYGF-YPWFRKTLSLIVT----------------

2rh1 NSTLNPIIYGF-YPWFRKTLSLIVTL---------------

4amj NSTLNPIIYGL-YPWFRKTLSLIV-----------------

4eiy NSTLNPIIYGLFYPWFRKTLSLIVTLRI-------------

3zpqA NSTLNPIIYGL-YPWFRKTLSLIVT----------------

4iar NSTLNPIIYGLFYPWFRKTLSLIVTLR--------------

# Supplementary Figure 1 Ramachandran Plot for TAAR13c

Energetically allowed regions for backbone dihedral angles ψ against φ of [amino acid](https://en.wikipedia.org/wiki/Amino_acid) residues in [TAAR13c](https://en.wikipedia.org/wiki/Protein_structure) are shown as contour map, color code see below. The backbone conformation of the final model was inspected by Ramachandran plot. Dihedral angles were in the allowed region for all but one residue. This outlier, Thr325 in the general psi–phi plot is located in the C-terminal, far away from the putative binding site and therefore does not affect our structure. The plot was generated by Rampage ^3^

# Supplementary Table 1 Interacting residues within the external and internal binding site

Shown are the residues, which are predicted by docking to interact with cadaverine (cad) and putrescine (put). Mutant TAAR13c is indicated by the amino acid exchange, e.g. D112A.

| *Wildtype/ Mutant* | *Interacting Residues*  *Cad Put* | |
| --- | --- | --- |
| TAAR13c | D112, L113, D202, T203, W269, F272 | D112, L113, T116,T203, T206, F207 W269, F273 |
| D112A | T116, D202, T206, F207, F272, F273, W269 | L113, T116, D202, T203, T206, F207, F272, W269 |
| D112E | L113, T116, D202, T206, F207, W269, F272 | L113, T116, T203, T206, F207, W269, F272 |
| D112N | L113, T116, D202, F272 | D202, T203, T206, W269, F272, F273 |
| D202A | T203, D112, T116, W269, F272, F209, F273 | D112, L113, W296, F272, F273 |
| D202E | D112, T116, T203, F207, W269, F273, | D112, L113, T203, T206, W269, F272 |
| D202N | W98, H108, T109, D112, F291, Y299 | R92, D112, S119, L192, Y288 Y299, F272, F291 |
| D279A | D112, T116, D202, T203, T206, W269, F272 | D112, T116, T203, T206, F207, W269, F272 |
| D279E | D112, L113, T116, D202, T203, T206, W269, F273, Y299 | T203, T206, F207, D112, L113, T116, W269, F272 |
| D279N | D112, L113, T116, D202, T203, T206, F207, W269, F272 | D112, L113, T116, T203, T206, F207, W269, F272 |

# References

1 Hussain, A. *et al.* High-affinity olfactory receptor for the death-associated odor cadaverine. *Proc Natl Acad Sci U S A* **110**, 19579-19584 (2013).

2 Zhang, J., Yang, J., Jang, R. & Zhang, Y. GPCR-I-TASSER: A Hybrid Approach to G Protein-Coupled Receptor Structure Modeling and the Application to the Human Genome. *Structure* **23**, 1538-1549 (2015).

3 Lovell, S. C. *et al.* Structure validation by Calpha geometry: phi,psi and Cbeta deviation. *Proteins* **50**, 437-450 (2003).
